# The Burden of Endometriosis on Women’s Lifespan: A Narrative Overview on Quality of Life and Psychosocial Wellbeing

**DOI:** 10.3390/ijerph17134683

**Published:** 2020-06-29

**Authors:** Luigi Della Corte, Claudia Di Filippo, Olimpia Gabrielli, Sabrina Reppuccia, Valentina Lucia La Rosa, Rosalia Ragusa, Michele Fichera, Elena Commodari, Giuseppe Bifulco, Pierluigi Giampaolino

**Affiliations:** 1 Department of Neuroscience, Reproductive Sciences and Dentistry, School of Medicine, University of Naples Federico II, 80131 Naples, Italy; claudifilippo@gmail.com (C.D.F.); olimpia.gabrielli3@gmail.com (O.G.); sabrinareppuccia@gmail.com (S.R.); giuseppe.bifulco@unina.it (G.B.); 2 Department of Educational Sciences, University of Catania, 951234 Catania, Italy; valarosa@unict.it (V.L.L.R.); e.commodari@unict.it (E.C.); 3 Health Technology Assessment Committee, A.O.U. Policlinico V. Emanuele, 95123 Catania, Italy; ragusar@unict.it; 4 Obstetrics and Gynecology Unit, Department of General Surgery and Medical Surgical Specialties, University of Catania, 95030 Catania, Italy; profmichelefichera@gmail.com; 5 Department of Public Health, University of Naples Federico II, 80131 Naples, Italy; pgiampaolino@gmail.com

**Keywords:** endometriosis, lifespan, quality of life, questionnaire, social relationship, economic burden

## Abstract

Endometriosis is a chronic, inflammatory disease affecting more than 170 million women worldwide and up to 10% of women of reproductive age. As a consequence of inflammatory reaction and infiltration of anatomic structures, endometriosis can cause “pain symptoms” including dysmenorrhea, dyspareunia, dyschezia, dysuria, and chronic pelvic pain. In this review, we summarized the impact of endometriosis on quality of life in all its aspects including sexual life, work, and social relationships. The data research was conducted using web-based search engines and/or various electronic research databases querying for all articles related to endometriosis and quality of life from the inception of the database up to February 2020. Endometriosis has not only physical but also psychological effects, causing depression, anxiety, and compromising social relationships. Furthermore, endometriosis negatively impacts sexual life and social relationships. At last, the economic burden of endometriosis should not be underestimated, both individually and for the community, as this pathology leads to a loss of productivity at work and large use of health resources. Thus, endometriosis-related symptoms control women’s lives compromising the quality of life in all aspects. In this review, we summarized the impact of endometriosis on various aspects of women’s lives.

## 1. Introduction 

Endometriosis is a chronic, and often progressive, inflammatory disease defined as the abnormal presence of endometrial tissue outside the uterine cavity [1]. Ectopic tissue reacts in the same way as the endometrium during the menstrual cycle. This tissue can form lesions on the ovaries, intestines, bladder and in the Douglas pouch as well as in pleura, lung parenchyma, and airways [2,3,4]. 

Endometriosis affects more than 170 million women worldwide [5] and up to 10% of women of reproductive age, with a higher prevalence in women with dysmenorrhea (40–60%), subfertility (21–47%), and/or pelvic pain (71–87%) [6]. 

The main etiopathogenetic theory is the retrograde menstruation theory, which has gained significant ground since it was first described in 1925 [7,8]. Along with this theory, other etiological factors have been related to the development of endometriosis such as immune dysfunction, genetic predisposition, environmental factors (dioxin and polychlorinated biphenyl) [9], and lifestyle risk factors including alcohol and caffeine [2,10,11]. 

An area of great interest among etiopathogenetic theories is the relationship between endometriosis and innate immunity. Recently, Karadadaş E et al. have shown that some complement factors are involved in the pathogenesis and probably in the progression of the disease; in particular, this study has highlighted the correlation between the complement protein C6 and endometriosis’s stage, opening the possibility to consider it as a potential biomarker for the diagnosis of endometriosis [12]. 

There are different types of endometriosis: superficial peritoneal implants, endometriotic ovarian cysts, and deep infiltrating endometriosis (DIE) [13,14]. The old definition of DIE is a form of endometriosis that penetrates more than 5 mm under the peritoneal surface [15]. However, the 5-mm definition permits the inclusion only of the deeper lesions, so it is preferable to define deep endometriosis, as adenomyosis externa or adenomyosis-like nodules; these lesions can involve the uterosacral ligaments, vagina, intestinal wall, rectovaginal pouch, ureter, and bladder [16,17]. It is estimated that the incidence of DIE is around 20% of women with endometriosis [18]. As a consequence of inflammatory reaction and infiltration of anatomic structures, endometriosis can cause “pain symptoms” including dysmenorrhea, dyspareunia, dyschezia, dysuria, and chronic pelvic pain [19,20]. The diagnosis of endometriosis is often overlooked, and patients undergo medical wandering, sometimes for several years, before being diagnosed and treated. Already by investigating the cardinal symptoms of the disease, the patient can be directed towards a diagnosis. However, it must be considered the existence of asymptomatic forms of endometriosis, that can be diagnosed only after laparoscopy investigation. The vaginal examination can be useful to search for painful hardening of the vagina, uterosacral ligaments, and/or torus uterine (place of insertion of uterosacral ligaments on the posterior surface of the uterus), as well as pain in uterine mobilization. Digital rectal examination is also essential in assessing rectovaginal septum nodules or nodules that infiltrate the rectal wall [21]. Transvaginal ultrasound has the greatest sensitivity and specificity in identifying ovarian endometriomas. The classic ultrasound features are a unilocular cyst with homogeneous low-level fluid echogenicity (frosted glass appearance) and poor or mild vascular flow. Pelvic ultrasound for deeply infiltrating endometriosis is more demanding and less sensitive [6]. Magnetic resonance imaging (MRI) has also been found to have high diagnostic accuracy above all in the diagnosis of deep endometriosis of the uterosacral ligaments (85 and 88%), vaginal endometriosis (77 and 70%), and endometriosis of the colorectal (88 and 92%) [22,23]. Biomarkers can be also taken into consideration in the endometriosis diagnosis but none of them have adequate reliability for clinical use. Therefore, laparoscopy remains the gold standard for definitive diagnosis of endometriosis [24]. The treatment options for endometriosis include hormonal therapies, to achieve a hypo-oestrogenic status (oral contraceptives, progestins, danazol, GnRH agonists), pain-relieving agents (nonsteroidal anti-inflammatories, opioids) or surgical removal of endometriotic implants [25,26]. There is no consensus on the first-line treatment, nevertheless, many authors believe that empirical medical therapies should be used in the first instance. Well-tolerated, low-cost, easily accessible options are steroidal anti-inflammatory drugs (NSAIDs), other analgesics (paracetamol and opioids), the combined oral contraceptive pill (OCP), traditional or newer progestins (medroxyprogesterone acetate, norethisterone, dienogest), and gonadotropin-releasing hormone (GnRH) [25,27,28]. However, medical treatment can be associated with various side effects and a possible recurrence of symptoms after cessation of intake. Laparoscopy is the gold standard for both definitive diagnosis and treatment: the goal is ablation or excision of all visible lesions to obtain the maximum effect on pain relief and increase fertility [29]. Nevertheless, surgery may be associated with peri- and postoperative complications and also confers a risk of recurrence of disease even in optimally resected patients [30]. Hysteroscopy has a very limited role both in the diagnosis and in the treatment of adenomyosis [31,32]. Medical treatment should be always restarted after surgery to reduce the risk of recurrence [25]. There is a growing body of research (both quantitative and qualitative) which documents the negative impact of endometriosis on quality of life, sexual function, employment, as well as psychological aspects of life [33]. Pain-related to endometriosis is the main responsible for the negative impact of the disease on quality of life. Pain could cause a deterioration of sleep quality, more perceived stress, lower activity levels and many psychological comorbidities such as anxiety and depression [34,35]. Besides, pain can impair sexual activity, with further negative consequences on psychological health, quality of life, and intimate relationships [34]. Not only intimate relationships, but all types of social relations are compromised with experienced loneliness as a result of social isolation [36]. Symptoms such as fatigue, mood swings, and severe bleeding lead to absenteeism or the inability to work for long hours. Taking time off from work has made some women feel guilty. Absences from work not only affect the individual and their family, but they have costs also for the entire country [37]. Endometriosis is, therefore, a pathology that can have an impact on all aspects of life with economic implications both individually and for the community: In this review, we summarized the impact of endometriosis on various aspects of women’s lives such as sexual life, social and partner relationships, work, and quality of life in all its aspects. 

## 2. Materials and Methods 

The data research was conducted using the following databases MEDLINE, EMBASE, Web of Sciences, Scopus, ClinicalTrial.gov, OVID and Cochrane Library querying for all articles related to endometriosis from the inception of the database up to February 2020. The studies were identified with the use of a combination of the following text words: endometriosis, quality of life, sex, cost, depression, anxiety, emotion, social relationship, economic burden, health care resource, questionnaire. The selection criteria of this narrative review included randomized clinical trials, nonrandomized controlled studies (observational prospective, retrospective cohort studies, case-control studies, case series), and review articles. A review of articles also included the abstracts of all references retrieved from the search. Conference papers and reviews and studies with information overlapping another publication were excluded. In the event of overlapping studies, we selected the most recent and/or most comprehensive manuscript. 

**Figure 1:**
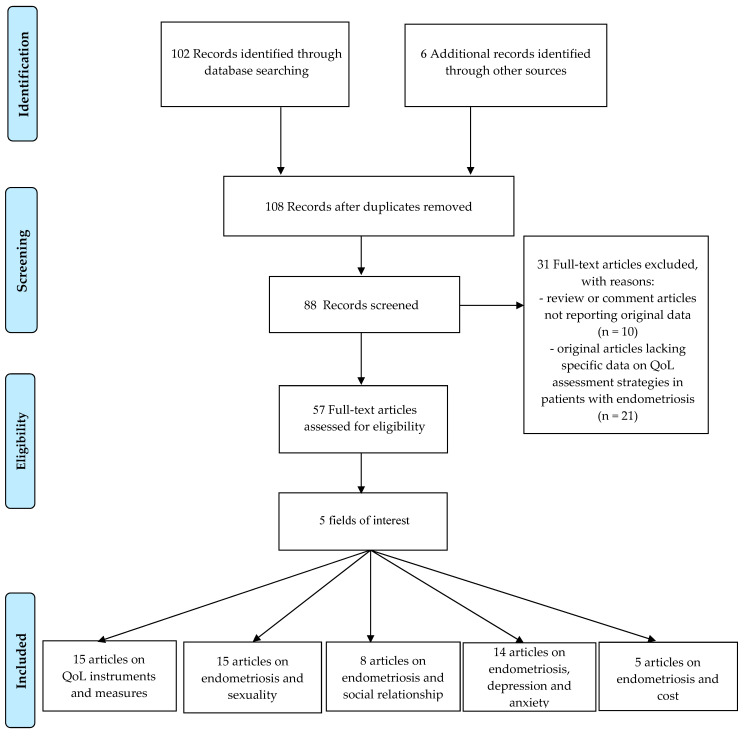
Flow diagram of the narrative review search.

We initially selected 108 studies from different databases: of these, only 88 records were screened. Of these, 57 studies were assessed for eligibility whereas 31 were excluded because of not reporting original data (10) and lacking specific data on quality of life assessment strategies in patients with endometriosis (21) (Figure 1). Titles and/or abstracts of studies retrieved using the search strategy and those from additional sources were screened independently by 2 review authors (L.D.C. and C.D.F.) to identify studies that potentially meet the aims of this nonsystematic review. The full text of these potentially eligible articles was retrieved and independently assessed for eligibility by other 2 review team members (O.G. and S.R.). Any disagreement between them over the eligibility of particular articles was resolved through discussion with a third (external) collaborator (P.G.). Two authors (C.D.F. and O.G.) independently extracted data from articles about study features and included populations, type of intervention (duration of therapy and drug posology), and outcomes. Any discrepancies were identified and resolved through discussion (with a third external collaborator where necessary).

According to World Health Organization (WHO), quality of life (QoL) is defined as a multidimensional construct of the individual perception of one’s position in life in the context of culture and value systems about goals, expectations, standards, and concerns [38]. Painful symptoms and infertility due to endometriosis, alone or combined, reduce QoL, impacting on all aspects of a woman’s life such as daily activities, employment and work productivity, mood, social and sexual relationships, family planning, and work productivity [39]. Several types of instruments are available to evaluate the multiple domains of QoL. 

## 3. QoL Instruments

### 3.1. QoL Instruments and Measures for Endometriosis 

Health-related quality of life (HRQoL) is a multidimensional concept that includes physical, psychological, and social aspects. Despite the importance of assessing the impact of endometriosis on QoL, there is still little consensus on which method to use. Several types of questionnaires have been proposed and used over the years (Table 1). 

**Figure 2:**
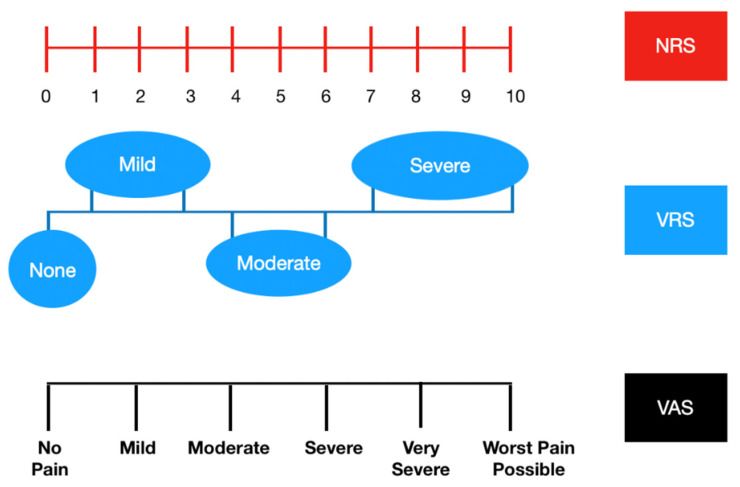
Three scales to evaluate the symptom “pain”: Verbal Rating Scale (VRS), Numerical Rating Scale (NRS), Visual Analogue Scale (VAS).

A recent review, conducted to assess the health-related QoL burden in women with endometriosis, has shown that the most commonly used QoL tools were the Short Form 36 (SF-36), the Short Form 12 (SF-12), and the World Health Organization Quality of Life Assessment-BREF (WHOQOL-BREF) [40]. The SF-36 consists of 36 items in eight domains: physical functioning, role-physical, bodily pain, general health, vitality, social functioning, role emotional, and mental health [41]. Women with endometriosis had significantly lower SF-36 scores than the general population, especially in the domains vitality, role-physical, and general health [42]. The SF-12 is made up of 12 items (taken from the SF-36) which produce two measures relating to two different aspects of health: physical and mental health [43]. The WHOQOL-BREF is a brief questionnaire comprising 26 items, including two items for overall QoL and general health, and another 24 items categorized in four domains (physical, psychological, social, and environmental health) [44]. The Nottingham Health Profile (NHP) assesses the physical, social, and emotional health of women with endometriosis. This questionnaire has two parts. Part I contains 38 objects (physical skills, pain, sleep, social isolation, emotional reactions, energy level). Part II, which is optional, provides a brief handicap indicator and considers the effect of health problems on employment, housework, personal relationships, social and sexual life, hobbies, and holidays [45]. Other less used tests are the Duke Health Profile and EuroQOL-5-dimension instrument (EQ-5D). The Duke Health Profile is useful in monitoring health. This questionnaire contains six health measures (physical, mental, social, perceived health, and self-esteem) as well as four dysfunction measures (anxiety, depression, pain, and disability) [46]. The EQ-5D is a generic instrument that includes five dimensions: mobility, self-care, daily activities, pain, and emotional well-being (depression or anxiety). Each item is scored based on a three-point scale, and the EQ-5D score is calculated by their sum, resulting in scores ranging from 0 (best possible status) to 10 (worst possible status) [47]. QoL can also be assessed using another questionnaire: the 15 Dimensional (15D) [48]. The questionnaire measures 5 levels of severity for each of the 15 dimensions (moving, seeing, hearing, breathing, sleeping, eating, speaking, eliminating normal activities, mental function, discomfort and symptoms, depression, anguish, vitality, and sexual activity). This questionnaire has proven to be well-validated, reliable, and sensitive [48]. Endometriosis has an impact also on work employment because women are unable to manage a full-time job because of difficulties in taking sick leave and because of workplaces that did not meet their needs; for this reason, it is important to evaluate also this domain of QoL [37]. The Health Related Productivity Questionnaire (HRPQ) is a 9-item measure of productivity, including absenteeism (work time missed, including household work) and presenteeism (reduced work effectiveness because of endometriosis, including household work) [49]. Subjective well-being (SWB) is a self-reported measure of well-being, commonly obtained by a questionnaire and proposed as a multiform construct comprising cognitive and affective components. The SWB can be studied with the Personal Wellbeing Index (PWI) which measures seven elements (standard of living, achievement in life, relationships, safety, connection with the community and future safety, as well as an element of overall life satisfaction) on a range from 0 (completely unsatisfied) to 10 (completely satisfied) [36]. To evaluate and quantify chronic pelvic pain, dyspareunia, and dysmenorrhea, several scales are available. One of the main tools for interviewing patients about their pain level is the Visual Analogue Scale (VAS) for five components: dysmenorrhea, dyspareunia, dyschezia, chronic pelvic pain, and dysuria. The VAS is considered the gold standard and consists of a 10 cm long horizontal line with the ends marked “no pain” and “worst imaginable pain” (Figure 2) [50]. Numerical Rating Scale (NRS) is a segmented numerical version of the VAS in which patients select, on a horizontal line or a bar, an integer from 0 to 10 (Figure 2); NRS better reflects the intensity of pain. Another scale used to evaluate different types of pain is the Verbal Rating Scale (VRS): with this type of scale, patients evaluate their pain intensity from absent (0) to severe (3) or from none (0) to very severe (5) (Figure 2) [51]. 

Dyspareunia is the symptom that most of all affects the quality of the sexual life of women with endometriosis: to evaluate it, there is DYSP diary. It is a single item that evaluates the dyspareunia during the last 24 h. The response options vary from Absent—0 (no discomfort during sexual intercourse), Mild—1 (I was able to tolerate the discomfort during sexual intercourse), Moderate—2 (intercourse was interrupted due to pain) to Severe—3 (I avoided sexual intercourse because of pain) [52]. To assess the quality of the sexual life of the patients, various questionnaires were used including the Questionnaire on Sexual Health Outcomes in Women (SHOW-Q), a complete questionnaire on women’s sexual function that assesses satisfaction, orgasm, desire of women, and the interference of disease with the sex [53]. A questionnaire widely used in various studies is the questionnaire of the Female Sexual Function Index (FSFI). It is made up of 19 items encompassing the six domains desire (items 1–2), arousal (items 3–6), lubrication (items 7–10), orgasm (items 11–13), satisfaction (items 14–16), and pain (items 17–19) representing the second part. Sexual dysfunction was defined as a FSFI-score < 26.55, based on published validations studies [54]. Female sexual dysfunction can be also evaluated with Female Sexual Distress Scale-Revised (FSDS-R) consisting of 13 elements to measure anxiety related to sex. The fixed-choice response format offers five increments: never, rarely, occasionally, often, and always. Sexual distress was defined as an FSDS-R score > 11 based on published validation studies. The higher the score, the greater the distress [55]. A disease-specific QoL measure is the Endometriosis Health Profile-30 (EHP-30)—a validated and reliable questionnaire that measures health-related QoL in women with endometriosis. The EHP-30 is a patient-reported outcome measure that represents the patient’s perspective about her experiences with the impacts of endometriosis. The EHP-30 is composed of a core questionnaire of 30 items, in addition to 6 modular parts containing 23 items. One of the 6 modular parts specifically addresses sexual intercourse, which includes questions about pain, guilt, worry, frustration, and avoidance associated with sexual intercourse. The reliability and validity of the EHP-30 have been assessed and affirmed [56]. A shorter version of EHP-30, more practical and suitable for clinical practice and also for research, was the Endometriosis Health Profile-5 (EHP-5). The EHP-5 is built in two parts: a 5-item core questionnaire about pain, control and powerlessness, emotions, social support, self-image, and a 6-item modular questionnaire about work-life, relation with children, sexual intercourse, medical profession, treatment and infertility. The response system consists of five levels ranged in order of severity: “never”,“rarely”, “sometimes”, “often”, and “always” [57]. All these questionnaires are used to evaluate how endometriosis affects the various aspects of patients’ life; however, it is not yet clear which one is the best. A recent review by Bourdel et al. shows that the two scales most frequently used are the SF-36 and EHP-30 and that the most validated scales were SF-36 and EQ-5D for general questionnaires and EHP30 and its abbreviated form EHP-5 for specific ones [45] (Table 1). 

### 3.2. Endometriosis and Sexuality 

Endometriosis-related symptoms can affect the sexual life of women with a decrease in the number and quality of coitus and compromising overall sexual activity, self-esteem, and sexual satisfaction [58]. The impairment of sex life is the main factor that compromises QoL of women with endometriosis, as shown in a market research survey conducted on 2753 women with symptomatic or asymptomatic disease [59]. The negative impact of endometriosis on sex life is mainly caused by dyspareunia, chronic pelvic pain, and psychological factors, mostly depression [60,61]. Endometriosis is associated with deep dyspareunia defines as pain or discomfort on deeper penetration, perceived in the vaginal canal or pelvic region [62]. The deep dyspareunia has a multifactorial etiology, including central sensitization, but it can be directly due to endometriosis-specific factors such as deep infiltrating endometriosis (DIE) [63]. Vercellini et al. carried out a study that compared women with recto-vaginal endometriosis (*n* = 100), peritoneal and/or ovarian endometriosis (*n* = 100), and a group of healthy controls (*n* = 100). The authors founded that women with endometriosis experienced more frequent and severe deep dyspareunia and consequent worse sexual functioning compared with controls (67, 53, and 26%, respectively). Instead, no statistically significant differences were observed between women with different localization of endometriosis (recto-vaginal or peritoneal/ovarian) [64]. However, Mabrouk et al., in a more recent observational study, founded that women with DIE experienced deep dyspareunia more frequently (85.2%) than women with OVA (isolated ovarian endometriosis) (70.9%) [58]. In addition, previous studies have correlated the presence of dyspareunia with the presence of DIE, specifically of the uterosacral ligaments resulting in a significant reduction in QoL and sexual function. Indeed, Ferrero et al. demonstrated that among subjects with deep dyspareunia, those with DIE of the uterosacral ligaments have the most severe impairment of sexual function. In particular, women with uterosacral ligament nodules had higher pain scores, a reduced number of intercourses per week, and a less satisfying orgasm and felt less relaxed and fulfilled after sex than the other groups [65]. These data show that DIE is the type of endometriosis most associated with dyspareunia and so with impaired sexual function [14]. When considering the relation between deep dyspareunia and sexual QoL (SQoL), it is fundamental to take into account potential confounders that can affect sexual function, such as superficial dyspareunia, other types of pelvic pain, psychological comorbidities, and concurrent pain diagnoses [60]. Shum et al. conducted a study in which showed that deep dyspareunia was associated with worse SQoL in women with endometriosis independently of other confounders [66]. As above mentioned, in addition to dyspareunia, the psychological state can also compromise sexual activity. Depression has been associated with impairment of SQoL in terms of sexual desire, sexual arousal, sexual cognition, and orgasmic functions [67]. Therefore, women with endometriosis perceived the frequency of sexual contacts significantly more often as “too low” than the control women (42.3 vs. 30.5% respectively; *p* < 0.001). There was also a significantly lower frequency in preliminary performance (“never” 31.3%, vs. 26.6% respectively; *p* = 0.003) [68]. Sexual dysfunction and deteriorating QoL appear to be related, as shown in the study by Montanari et al. that have assessed sexual function with SHOW-Q scores and health-related QoL through SF-36 in particular in women with DIE. The authors founded a significant correlation between the SF-36 scores and the SHOW-Q scores (*p* < 0.0001). The average values obtained on the SHOW-Q scales showed poor sexual function (average total SHOW-Q score 56.38–22.74). Satisfaction was the most affected dimension (average satisfaction score 55.66–34.55), followed by orgasm (average orgasm score 56.90–33.77). Moreover, in this study, it was highlighted that among women with DIE, only those with vaginal lesions significantly have a significant impact on sexual function [69]. As demonstrated by a large and recent study, sexual health is a highly important aspect of quality of life: sexual health should be part of any clinicians’ assessments and the improvement of SQL should be considered as a purpose of treatment [70]. Therefore, it is important to highlight the potential of surgical and medical treatment as an answer to improving sexual symptoms and quality of life. In a review of Fritzer et al., 69 articles were evaluated regarding the removal of endometriotic implants and the effects on dyspareunia after surgery. All included studies showed significant improvement (*p* < 0.05) in pain during intercourse after the surgical excision of endometriotic lesions. A reduction in dyspareunia and an improvement in sexual activity are observed twelve months after complete excision of endometriosis. Besides, sexual satisfaction has increased, and sexual problems have decreased significantly. Surgical excision of endometriosis is a feasible and good treatment option to relieve pain and improve the quality of sexual life in symptomatic women with endometriosis [71]. Even medical therapy, in particular progestin, can improve the symptoms of patients [20]. The comparison between the two therapeutic strategies shows that both are effective in relieving endometriosis-associated deep dyspareunia, although with a different temporal trend [72]. 

### 3.3. Endometriosis and Social Relationships 

There are many pieces of evidence about the negative impact of endometriosis on relationships. An international multicenter survey founded that 50% of 3216 women, invited to participate in the study, reported that endometriosis had affected their relationships, causing a couple split in 10% of cases [73]. The evaluation conducted in a recent study on subjective wellbeing (SWB) and health-related quality of life (HRQoL) showed that women with endometriosis have reported a negative impact on relationships, in particular for the lack of understanding and support from others [36]. In addition, previous studies have shown that women feel ashamed of their condition and as a result feel unable to discuss their health with their employer, colleagues, friends, and family [74]. This can lead to the fact that the women felt isolated and alone with endometriosis, as shown in a narrative review on the social and psychological impact of living with endometriosis [34]. It was also highlighted that sometimes the consideration of the effect of the disease on the quality of life is not taken into consideration even by clinicians with consequent compromise in the patient’s medical relationship. In a qualitative study, it has been reported that women highlighted negative experiences with health care clinicians, not receiving support from them [39]. Moreover, most clinicians assessed themselves not adequately trained to understand and provide psychosocial care for this group and many found it not necessary to do so [75]. The major and most frequent negative effect of endometriosis is on intimate relationships. Dyspareunia harms sex and intimacy for couples. Fagervold et al. found a correlation between dyspareunia and negative impacts of endometriosis on relationships (*p* = 0.004) [76]. However, a cross-sectional qualitative study, the ENDOPART study, demonstrated that also general fatigue, a reduction in sex drive due to drugs, a weak mood, bleeding during and/or after sex, and problems in attempts pregnancy have an impact on the relationship. In particular, 18 out of 22 couples reported that endometriosis had somehow influenced their plans for having children. Therefore, implications also occur for male partners in many life domains, including planning for having children, working lives, household income, support roles, and has a substantial influence on men’s emotions [77]. The implications for the stability of the couple relationship are easily understood. 

### 3.4. Endometriosis, Depression and Anxiety 

Endometriosis is a problematic disease in which symptoms control women’s lives also causing important psychological effects [78]. Cavaggioni et al. reported that women with endometriosis had a higher prevalence of any depressive (18.9 versus 9.3%) and anxiety disorders (29.7 versus 7.0%) than controls [79]. This thesis was also demonstrated by a longitudinal study that analyzed 10,439 women with endometriosis and 10,439 controls. All enrolled women had no history of any psychiatric disorder before registration. The study showed that women with endometriosis had an increased risk of developing major depression (hazard ratio [HR]: 1.56, 95% confidence interval [CI]: 1.24–1.97) and any depressive disorder (HR: 1, 44, 95% CI: 1.25–1.65) and anxiety disorders (HR: 1.44, 95% CI: 1.22–1.70) compared to those without endometriosis [80]. Overall, most of the literature agrees to consider depression, anxiety, and emotional distress more frequently in women with endometriosis than in a healthy population [34]. There is still no agreement on the origin of this evident correlation. Some authors showed that depression and anxiety may be the result of the experience of pelvic pain itself rather than of endometriosis since the rate of these psychological disorders was not different between women with endometriosis-related pelvic pain and those with pelvic pain of another nature [81,82]. However, even when rates of depression and anxiety appeared to be higher for women with endometriosis-related pain, the causal direction could not be identified [83]. Anyway, a mutual relationship between pelvic pain and emotional function has been highlighted; anxiety and depression increase pain perception, and pain can compromise the psychological state in a vicious circle [79]. 

Depression negatively affects different aspects of quality of life such as relationships, sex, work, and even sleep quality. Poor sleep quality (reduction of total sleep time, frequent awakenings, and difficulty falling asleep) can in turn negatively affect the ability to perform daily functional activities in women with endometriosis [84]. Moreover, poorer and poorer sleep can lead to an exacerbation of pelvic pain and this negatively affects the quality of life [85,86,87]. The association between sleep quality and depression is probably two-way, with poor sleep quality worsening mood and depression which in turn affects sleep [88,89]. There are few data in the literature about the influence of medical and surgical treatments on psychiatric comorbidities related to endometriosis. Available studies on both hormone and surgical therapy have shown promising results in improving psychiatric symptoms. However, further studies are necessary [90]. 

### 3.5. “Costs” of Endometriosis 

The economic burden of endometriosis has been well documented in the literature. A prospective, multicenter survey conducted in 10 European countries (EndoCost study) demonstrated that the average annual total cost per patient with endometriosis in 2008 was almost €10,000, including health care as well as loss of productivity costs [85]. The most important items of health care costs were surgery (29% of health care costs), monitoring tests (19%), hospitalization (18%), and physician visits (16%). The annual economic burden of endometriosis, including direct health care costs and indirect productivity loss, was estimated to be $22 billion in 2002 and $69.4 billion in a 2009 follow-up study, a substantial apparent increase in costs attributed to endometriosis over time [91]. Fuldeore et al. (2015) found that in the US, annual healthcare resource utilization and costs were highest in the first year following an endometriosis diagnosis, costing $13,199 compared with $6041 in the year before diagnosis and $6720 in the following year. Additionally, in the five years before an endometriosis diagnosis, costs were $7028 higher among patients with endometriosis compared with matched controls without endometriosis [92]. Soliman et al. evaluated, in a retrospective cohort study, direct health care utilization and costs among women with endometriosis in comparison with age-matched controls in a U.S. Medicaid population. Direct health care resource utilization (HCRU) during the 12-month follow-up period was significantly higher for endometriosis cases compared with controls in all measured categories: hospital admissions, emergency room visits, mean office visits, and finally prescription claims. The highest expenditure category for endometriosis patients was inpatient admissions ($5,785) followed by other outpatient services ($4363) and outpatient prescriptions ($2,096). The mean ± SD total health care costs were higher for patients with endometriosis, $13,670 ± $29,843, compared with those without endometriosis, $5,779 ± $23,614 [93]. The same authors of the above-mentioned review have also shown that, in employed women with endometriosis, as a consequence of productivity loss of 6.3 h per week, the total loss per person is approximately $10,177.54 per year [49]. 

## 4. Conclusions

Endometriosis is a chronic disease affecting a large portion of the world’s female population of childbearing age. The quality of life is strongly influenced by this pathology: women suffer from dysmenorrhea as well as chronic pelvic pain and this affects work, leisure, and social and love relationships. Pain-related to endometriosis also affects the psychological aspect, compromising the quality of sleep, making women anxious and depressed. 

The impact of endometriosis on sexual life is huge: dyspareunia is one of the cardinal symptoms of the pathology. This symptom reduces the frequency of sexual intercourse, worsens the QoL and the SQoL with a negative impact also on the couple’s life. The costs of endometriosis should not be underestimated, both in terms of treatment and loss of productivity of the woman due to the disease. 

It can be concluded that endometriosis is a pathology that affects all aspects of women’s lives and that thus, it must be treated with a multidisciplinary vision that includes not only a medical approach but also psychological, work, and economic support. 

## Figures and Tables

**Table 1:** Endometriosis related questionnaires.

Questionnaires	Number of Questions	Domains	Scores	What Does it Assess?	References
Short Form 36 (SF-36)	36 items	Eight domains: vitality, mental health, bodily pain, general health perceptions, physical, role-physical, social and role emotional functioning	Scores range from 0 to 100, with higher scores indicating better QoL	QoL	Ware JE Jr et al. [40] (1992)
Short Form 12 (SF-12)	12 items	Eight domains: vitality, mental health, bodily pain, general health perceptions, physical, role-physical, social and role emotional functioning	Scores range from 0 to 100, with higher scores indicating better QoL	QoL	Gandek B et al. [41,42,43] (1998)
Nottingham Health Profile (NHP)	Part I: 38 items Part II: 7 items	Part I: six domains (physical abilities, pain, sleep, social isolation, emotional reactions and energy level) Part II: seven domains (problems on employment, jobs around the house, personal relationships, social and sex life, hobbies and holidays)	All questions have only yes/no answer options and each section score (maximum 100) is weighted.	QoL	Bourdel N et al. [44] (2019)
World Health Organization Quality of Life Assessment-BREF (WHOQOL-BREF)	26 items	Four domains: physical, psychological, social, and environmental health	Scores for each item range from 1 to 5, with the highest score indicating the best QoL	QoL	The WHOQOL Group [45] (1998)
Duke Health Profile (DUKE)	17 items	Six health measures (physical, mental, social, perceived health, and self-esteem) and four dysfunction measures (anxiety, depression, pain, and disability).	Scores for each measure range from 0 to 100. For health measures high score = good health; for dysfunction measures high score = poor health	QoL	Parkerson GR Jr et al. [46] (1990)
Euro QOL-5-dimension instrument (EQ-5D)	5 items	Five dimensions: mobility, self-care, daily activities, pain, and emotional well-being (depression or anxiety) + EQ Visual Analog Scale (EQ-VAS) on health status	Scores expressed initially as a 5-digit number can be converted into a single weighted index score that describes the patient’s health state. + VAS with a grade ranging from 0 (the worst possible health status) to 100 (the best possible health status)	QoL	EuroQol Group [47] (1990)
15-Dimensional (15D)	15 items	Breathing, mental function, speech (communication), vision, mobility, usual activities, vitality, hearing, eating, elimination, sleeping, distress, discomfort and symptoms, sexual activity, and depression.	The single index score uses a 0–1 scale (1 corresponds to no problems on any dimension)	QoL	Sintonen H [48] (2001)
Health Related Productivity Questionnaire (HRPQ)	9 items	Four sections evaluating also absenteeism (missed work hours at paid employment, in the home, or at educational activities) and presenteeism (reduced effectiveness during any work that is attempted) + an optional section for younger age-group patients and those pursuing education	Specific algorithm	Work productivity	Soliman AM et al. [49] (2017)
Personal Wellbeing Index (PWI)	8 items	Seven domains: standard of living, achievement in life, relationships, safety, connection with the community, future safety, overall life satisfaction	Score range from 0 (completely dissatisfied) to 10 (completely satisfied)	SWB	Rush G et al. [35] (2018)
DYSP diary	Single items	Intensity of dyspareunia during 24 h	Absent (no discomfort during SI), Mild (tolerable discomfort), Moderate (SI interrupted), Severe (SI avoided)	Dysp	Pokrzywinski R et al. [50,51,52] (2020)
Questionnaire on Sexual Health Outcomes in Women (SHOW-Q)	12 items	Sexual satisfaction, orgasm, sexual desire, and pelvic problem interference with intercourse	All items were scored on a scale from 0 to 100; higher scores represent better sexual function except for the fourth domain. The overall score corresponds to an average of the 12 articles	SQoL	Learman LA et al. [53] (2008)
Female Sexual Function Index (FSFI)	19 items	Six domains: desire, subjective arousal, lubrication, orgasm, satisfaction, and pain	Each domain is scored from 0 to 6: higher scores indicate better sexual function. Total score of the questionnaire ranges from 2 to 36.	SQoL	ter Kuile MM et al. [54] (2006)
Female Sexual Distress Scale-Revised (FSDS-R)	13 items	Sexual distress	Every item requires an answer that is rated from 0 (never) to 4 (always). The total score, ranging from 0 to 48	SQoL	Derogatis L et al. [55] (2008)
Endometriosis Health Profile-30 (EHP-30)	Core instrument: 30 items Modular section: 23 items	Core items: pain, control and powerlessness, emotional well-being, social support, and self-image. Modular items: work, relationship with children, sexual relationship, feelings about medical profession, feelings about treatment, feelings about infertility	Each scale is standardized on a score ranging from 0 to 100, where the lowest score represents the best health status.	QoL	Jones G et al. [56] (2004)
Endometriosis Health Profile-5 (EHP-5)	11 items	Core questionnaires (pain, control and powerlessness, emotions, social support, self-image) and modular questionnaires (work-life, relation with children, sexual intercourse, medical profession, treatment and infertility	Total score ranging on a scale from 0 (indicating best possible health status) to 100 (indicating worst possible health status).	QoL	Jones G et al. [57] (2004)

QoL: quality of life; SI: sexual intercourse; Dysp: dyspareunia; SBW: subjective well-being; SQoL: sexual quality of life.
